# Scratch on Polymer Materials Using AFM Tip-Based Approach: A Review

**DOI:** 10.3390/polym11101590

**Published:** 2019-09-29

**Authors:** Yongda Yan, Shunyu Chang, Tong Wang, Yanquan Geng

**Affiliations:** 1 Key Laboratory of Micro-systems and Micro-structures Manufacturing of Ministry of Education, Harbin Institute of Technology, Harbin 150001, China; yanyongda@hit.edu.cn (Y.Y.); changshunyu@163.com (S.C.); wtong0211@163.com (T.W.); 2 Center for Precision Engineering, Harbin Institute of Technology, Harbin 150001, China

**Keywords:** atomic force microscopy, TBN method, scratching, polymer materials

## Abstract

As a brand new nanomachining method, the tip-based nanomachining/nanoscratching (TBN) method has exhibited a powerful ability at machining on polymer materials and various structures have been achieved using this approach, ranging from the nanodot, nanogroove/channel, bundle to 2D/3D (three-dimensional) nanostructures. The TBN method is widely used due to its high precision, ease of use and low environmental requirements. First, the theoretical models of machining on polymer materials with a given tip using the TBN method are presented. Second, advances of nanostructures achieved by this method are given, including nanodots/nanodot arrays, a nanogroove/channel, 2D/3D nanostructures and bundles. In particular, a useful approach called the ultrasonic vibration-assisted method introduced to integrate with TBN method to reduce the wear of the tip is also reviewed, respectively. Third, the typical applications of the TBN method and the nanostructures achieved by it are summarized in detail. Finally, the existing shortcomings and future prospects of the TBN method are given. It is confirmed that this review will be helpful in learning about this method and push the technology toward industrialization.

## 1. Introduction 

Nanotechnology, genetic engineering and intelligent technology are known as the “the most important three techniques of the 21st century”, among which, the rapid development of nanotechnology has brought us to the nano age [1,2,3,4]. Micro/nano manufacturing technology has been widely used in the environment, energy, biology, medicine, national defense and other fields, playing an increasingly important role in promoting national development and social progress [5,6,7,8,9,10,11]. Up until now, nanopatterns have exhibited huge potential applications in the areas of nano-grating sensor, nano optical, surface-enhanced Raman scattering (SERS) and so on [12,13,14,15,16,17,18,19,20,21,22,23]. Thus, how to achieve more complex nanostructures with high accuracy has become a hot issue. At present, various relatively mature techniques have been applied to fabricate nanostructures, mainly including focused ion-beam lithography (FIB) [24,25,26], electron-beam lithography (EBL) [27,28,29,30], ultra-violet lithography (UV lithography) [17,31,32,33,34] etc. However, various factors limit the widely use of these techniques in fabricating nanostructures, ranging from low resolution, high-cost of the equipment, relatively high demand for operation environment to a limited range of materials that can be processed. Therefore, a brand new method with a low-cost, ease of use, high accuracy and no need of vacuum environment is needed. The TBN nanofabrication method is born out of the development of atomic force microscopy (AFM).

AFM was first invented to characterize the surface morphology of the sample in 1986 [35]. However, in recent years it has been demonstrated to be a powerful nanomachining approach due to its properties of low-cost, nano-scale resolution, low environmental requirement and high accuracy, known as the TBN method [36,37]. The TBN method has exhibited a potential feasibility in fabricating various materials, ranging from polymer, metal to semiconductor [38,39,40,41,42,43,44]. In spite of this, rapid wear can be observed when using the TBN method to scratch on samples with a large normal load due to a relatively large hardness of the material [45]. Thus, other energies such as thermal, chemical and ultrasonic are integrated with the TBN method to reduce the wear of the tip and extend the service life of the tip [36,46,47]. Among them, the ultrasonic vibration-assisted approach is widely used in reducing the applied actual force between the tip and the sample so as to extend the service life of the tip, as well as to enlarge the depth of the structure effectively [2,48,49,50,51,52].

Polymer materials are widely used in the fields of the Micro/Nano-Electro-Mechanical System (MEMS/NEMS) technique, such as optical mask, flexible electronic device, nanosensor and nanofluidic due to its relative low-cost, good light transmittance and excellent biocompatibility [53,54]. Among them, the most important application of polymer film is as the resist of etching [52,55]. When scratching nanostructures on hard sample such as silicon or silicon dioxide, an extreme wear of the tip can be observed due to a relatively large hardness of the sample. The solution of above problem is using the TBN method to fabricate nanostructures on polymer resist film first, and later the etching approach like reactive ion etching (RIE) is utilized to transfer the nanopatterns to the hard substrates, such as semiconductor materials like silicon [56,57] and quartz [44,58,59]. Up to now, a lot of nanopatterns have been achieved on polymer materials using the TBN method [60,61], including nanodots/nanodot arrays, nanogroove/channel, bundles, 2D/3D nanostructures. One point that merits attention is that the mechanical remove process of polymer materials based on TBN method keeps the normal load constant so as to guarantee the accuracy of the machined nanostructures. However, many scholars were committed to applying the obtained nanostructures on polymer materials using the TBN method for preparations of industrial production. Although a lot of applications have been achieved in the fields of nanooptics, nanofluidic, nano-electronic devices and so on [62,63], more and more novel applications needed to be discovered in the future so that the TBN method can be used in industrial engineering as early as possible. Moreover, AFM exhibits a huge potential in the development of polymer technology, especially for the emerging field of “polymer brushes”, which is a technique that grafts polymers on to solid substrates [64] and has been applied in the field of sewage purification [65], adsorption of charged biomolecules [66], lubrication [64], adhesion [67], colloidal stability [68] and biotechnology [69]. However, there is also a problem that the desorption of chains during and after the brush creation remains a common phenomenon by the existing method [68], and therefore, a reliable approach is required. As is widely known, polymer pen lithography (PPL) as an important component of dip-pen nanolithography (DPN) based on AFM, which has been demonstrated to be a powerful tool in polymer molecules′ deposition [70]. With the applicable inks extending from metal to polymer in the past 20 years, the DPN method has been developed into as a tool for creating new materials, especially for polymers [70]. In conclusion, various techniques based on AFM are sure to make great contributions in the field of “polymer brushes” in the days to come.

Thus, in this paper, a review of recent advances of scratching polymer materials using TBN methods is given, which includes many current aspects, from theory to experiment and from advantages to shortcomings. First, theoretical models of the scratching process on polymer materials are reviewed. Then, current situation of the development in nanostructures fabricated on polymer materials by the TBN method is summarized in detail. Moreover, a summary about the applications of TBN method and the nanostructures achieved by it are also conducted. Finally, an overview of the existing deficiencies and the future directions of development is given. 

## 2. Theoretical Modeling of the Scratching Process 

### 2.1. Existing Theoretical Models of the Scratching Process

**Figure 1:**
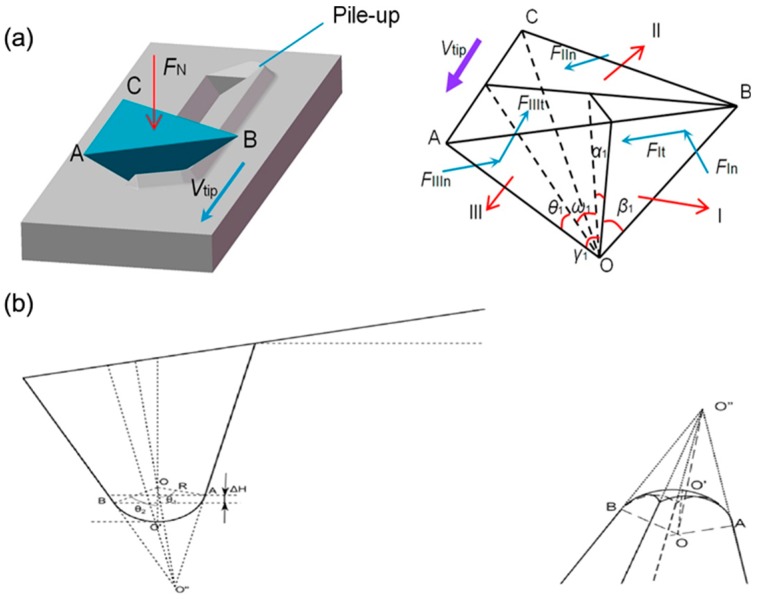
A simplified model of the probe: (**a**) the tip is modified as a triangular pyramid when scratching on polycarbonate (PC) bulk sample and (**b**) the tip is modified as a rectangular pyramid with a spherical apex when scratching on poly (methyl methacrylate) (PMMA) film [71,72].

The scratching process on polymer materials using the TBN method is affected by several factors, including the normal load applied on the sample surface, the friction between the tip and the sample, the elastic recovery of the material after nanomachining and the height of the pile-up formed owing to the accumulation of material etc. Therefore, it is necessary to establish a theoretical model to predict the influence of these parameters on nanoscratching process. Exciting results have been achieved during the past few years. A typical theoretical model was established by Geng et al. [71] to study the relationship between above factors and the depth of nanogroove during the process of scratching a nanogroove, which takes into consideration the effects of the normal load, scratching velocity, height of pile-up, friction between the tip and the sample, elastic recovery of the polymer material and probe geometry used in the scratching tests. In this model, in order to estimate the scratching depth of nanogroove, the normal load and lateral force applied on the tip in Figure 1a were first calculated and could be expressed as follows [71]:

(1) $$\{\underset{F_{N}}{\to }=\iint \left(\sigma_{f}\hat{n}\cdot \hat{z}+\mu_{a}\sigma_{f}\hat{t}\cdot \hat{z}\right)dA\hat{z}$$

(2) $$\underset{F_{V}}{\to }=\iint \left(\sigma_{f}\hat{n}\cdot \hat{v}+\mu_{a}\sigma_{f}\hat{t}\cdot \hat{v}\right)dA\hat{v}$$

where, $F_{N}$ is the normal force applied on the tip and $F_{V}$ is the lateral force and $\sigma_{f}$ is the flow stress during the scratching process. $\hat{n}$ is the normal unit vector perpendiculars to the surface of the probe and in the oblique upward direction. $\hat{z}$ is the unit vector in the vertical direction and $\mu_{a}$ is the adhesive friction coefficient. Moreover, $\hat{t}$ is the tangential unit vectors, which is in the opposite direction of the projection of the tip when moving on the surface of the probe. dA is the unit area of the contact field between the tip and the polymer sample surface. $\hat{v}$ is the unit vector in the lateral direction along the moving tip.

The verification experiments were conducted on a polycarbonate (PC) bulk sample, and 10 normal forces ranging from 23.3 to 133.8 μN and 13 scratching velocities ranging from 5 to 200 µm/s were used to study the relationship between the applied normal force, nanoscratching velocity, the height of pile-up and the fabricated depth of nanogroove. Finally, nanogrooves with desired depths are obtained on the surface of the PC sample by setting the parameters to suitable value under the guidance of this model. Results showed that the depths of nanogrooves fabricated by the TBN method in experiments exhibit a good fit with the expected depths predicted by this model. Generally, the model in this work provides an approach to make the dimension of nanogroove controllable so that we can achieve an ordered nanogroove easily using the TBN method on polymer materials. The sample used in the above work is a bulk sample, thus, the extreme wear of the tip could be neglected owing to the evident disparity between the probe and the polymer substrate. However, an extreme wear of the tip will occur when using the TBN method to scratch on a polymer thin-film spin-coated on a hard substrate resulting from the direct contact between the tip and the substrate if the thin film is penetrated through by the tip under a relatively large normal load. Therefore, in order to rise the above limitations of the model, Zhou et al. [72] established a mathematical model for the process of scratching nanogrooves on a poly (methyl methacrylate) (PMMA) thin film using the TBN method. This model takes the parameters affecting the fabrication process into consideration, including the friction between the tip and the sample, the elastic recovery of the polymer material, the height of the pile-up and the flow stress of the sample. Another different point between this model and that proposed by Geng et al. [71] is the tip geometry used in the model, as shown in Figure 1. The tip utilized in the model proposed by Geng et al. [71] was regarded as a triangular pyramid, while, the tip used in model established by Zhou et al. [72] was simplified as a rectangular pyramid with a spherical apex. Moreover, the calculation of the model proposed by Zhou et al. [72] is dependent on the relationship between the machined depth and the height of the apex of the probe [73]. To achieve good control of the depth of the nanogroove, the normal load and tangential force applied on the unit contact area between the tip and the polymer sample were described as Equation (3) [74] and Equation (4) [74]:

(3) $$\underset{dF_{N}}{\to }=\sigma_{f}dA\hat{n}$$

(4) $$\underset{dF_{t}}{\to }=\mu_{a}\sigma_{f}dA\hat{t}$$

where, $\sigma_{f}$ is the flow stress during the scratching process and $\mu_{a}$ is the adhesive friction coefficient. $\hat{n}$ is the normal unit vector and $\hat{t}$ is the tangential unit vectors.  dA is the unit area of the contact field between the tip and the polymer sample surface. Moreover, the total height of the contact area between the AFM tip and the polymer sample was also calculated by a simple method when the tip is cutting through the polymer thin-film, which is shown in Equation (5) [72]:

(5) $$H_{total}=H_{PMMA}+\;H_{pile-up}$$

where, $H_{total}$ is the total height of the contact area between the AFM tip and the polymer sample and $H_{PMMA}$ is the thickness of PMMA thin-film. $H_{pile-up}$ is the height of pile-up accumulating on the side of the nanogroove during scratching process.

This model shows an outstanding capacity in predicting the normal force that needs to be applied on the sample surface so that the tip can just penetrate the polymer thin film without contacting with the hard substrate directly. Under the guidance of this model, several nanochannels were fabricated on PMMA thin films with various thicknesses to provide proofs that the tip just cut through the thin film. These results were achieved by regulating the normal load to a suitable value using tips with different radii. This indicates that this model can be used to predict the normal force required to obtain a nanogroove using a tip with a given radius just cutting through the sample when nanoscratching on polymer thin film. Therefore, this model makes a great contribution to reducing the wear of the tip when scratching on a polymer thin-film spin-coated on a hard substrate, such as silicon and silicon dioxide. In conclusion, there is very little study about the theoretical modeling of the scratching process so far, which needs to be further studied in the future.

### 2.2. Study of the Elastic Recovery of Polymer Materials in Scratching Process

**Figure 2:**
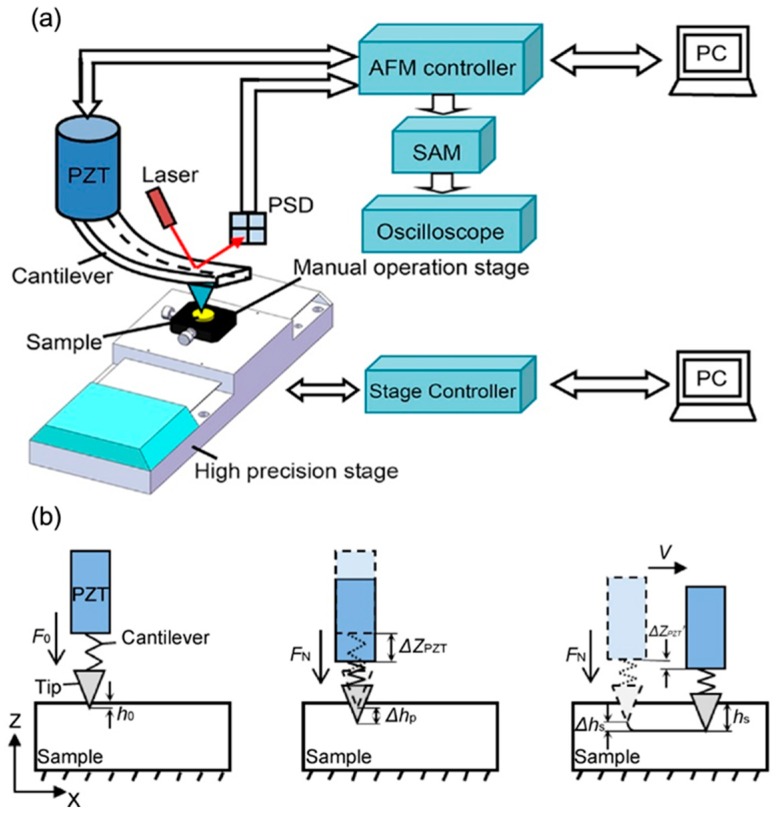
Schematic diagram of measuring elastic recovery of material: (**a**) modified atomic force microscopy (AFM) system based on the TBN method and (**b**) the position of the tip when using a light normal load to scratch the PC bulk sample [76].

When scratching a nanogroove on polymer materials using TBN method, the depth of the nanogroove will decrease after scratching due to the elastic recovery of the polymer materials. This phenomenon happens owing to the unique property of polymer materials, which is known as high viscoelastic. Thus, it is necessary to study the mechanism of elastic recovery in scratching polymer materials by the TBN method. However, most scholars were focusing on the elastic recovery of nanodots fabricated on polymer materials by the nanoindentation method [75], and the study of elastic recovery in nanogroove is relatively less. Geng et al. [76] established a constitutive model to estimate the elastic recovery of a nanogroove after scratching. The schematic diagram of this method is shown in Figure 2. Figure 2a is the modified AFM system used in this approach and Figure 2b is the position of the tip when using a light normal load to scratch the PC bulk sample. The elastic recovery of the sample was obtained by comparing the machined depth and the measured depth. The machined depth in this work was achieved by observing the vertical signal change of the piezoceramic tube (PZT) in a modified AFM system using an oscilloscope. The measured depth was achieved by imaging the groove using AFM tapping mode. The verified experiment was conducted on a PC bulk sample, which demonstrated the feasibility of this method in estimating the elastic recovery of polymer materials. Moreover, the results also presented that the sample elastic recovery was affected by the speed of scratching, while almost no influence was caused by the applied normal load. This is a simple and easy method to estimate the elastic recovery of polymer materials in nanoscale. Using this method, we can achieve desired nanogrooves in high accuracy by predicting the elastic recovery of the sample in advance. In spite of this, more theoretical study is required so as to fabricate nanostructures with higher accuracy and make the dimension of the machined structures controllable.

## 3. Nanopatterns Fabricated by the Tip-Based Nanomachining/Nanoscratching (TBN) Approach 

Nanodots, nanogroove/channel, bundles and 2D/3D nanostructures are widely used in high density storage, nanofluidic, nano-electronics and nano-optics. Moreover, polymer material exhibits a huge potential application for their relative low-cost, better translucency and excellent biocompatibility. Thus, many scholars proposed various methods to fabricate nanopatterns on polymer materials. The following sections will present the status of fabricating nanopatterns on polymer materials using the TBN method.

### 3.1. Fabrication of Nanodots/Pits

Nowadays, nanodot arrays are widely used in the preparation of quantum dots, nano-optics sensors and surface-enhanced Raman scattering (SERS) substrate. Nanopits exhibit huge potential application in data storage.

**Figure 3:**
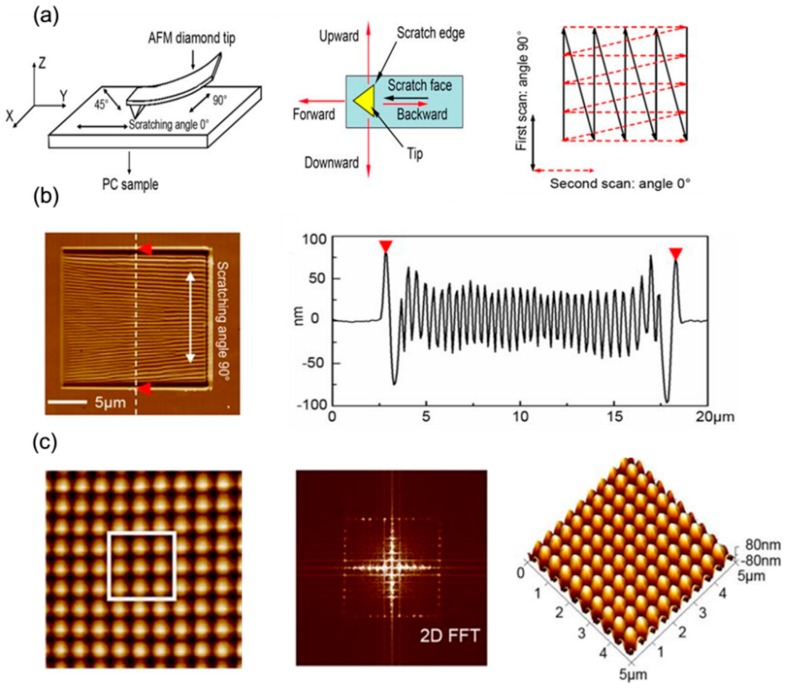
Schematic diagram of the formation process of nanodot arrays: (**a**) trajectory of the tip when making a two-step scanning at 90°. (**b**) the 2D image of bundles after the first-step scanning and its cross-section. (**c**) 2D/fast Fourier transform (FFT)/3D image of nanodot arrays when scratching at 90° and 0° [80].

**Figure 4:**
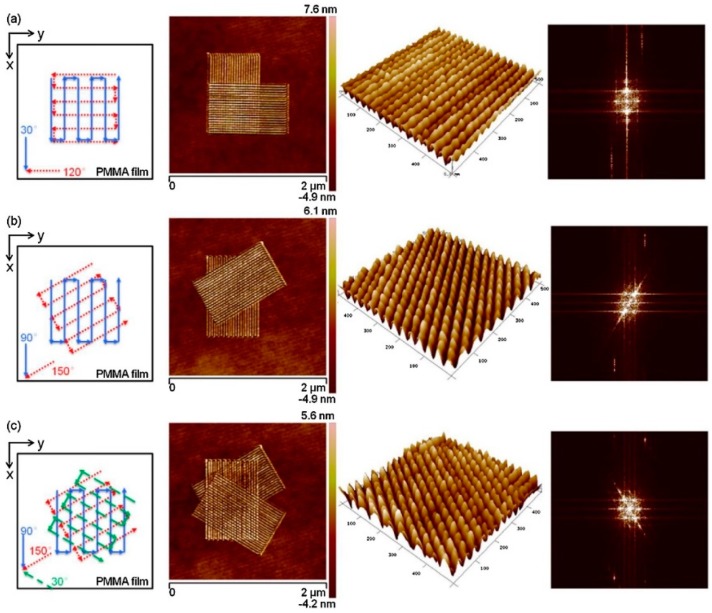
Nanodot arrays fabricated by dynamic plowing lithography (DPL) method: (**a**) checkerboard nanodot arrays fabricated by two-step scanning at 30° and 120°. (**b**) Diamond-shaped nanodot arrays fabricated by two-step scanning at 90° and 150°. (**c**) Hexagonal nanodot arrays fabricated by three-step scanning at 30°, 90° and 150° [81].

There are several nanomanufacturing methods to fabricate nanodot arrays. Up to now, TBN method have been proved to be a feasible method to fabricate nanodots arrays [77]. There are two of the main methods based on the TBN technique utilized to fabricate nanodots arrays. One of them is the simple nanoindentation process [75,78,79]. In this method, the size of nanodot depends on the radius of tip, the normal load and the properties of the polymer materials. A high aspect ratio of nanodot less than 100 nm can be obtained by controlling a small normal load using a sharp tip to penetrate into the polymer materials. Another method is scratching on polymer materials. By using this method, the dimension of the nanodot is mainly controlled by the pitch of the nanoscratching process, the properties of the polymer materials and the normal load. Sun et al. [80] conducted a single zigzag scan on polycarbonate (PC) surface to fabricate bundles structure. In their study, the influences of the scan angle, normal load and feed between two scratching trajectories on the bundle structures obtained were studied. Based on the formed bundles, some scholars change the scan angle to make a second-scan on the pre-formed bundles structures. Nanodots can be formed by the overlapping of the bundles obtained during the first- and second-scan. As shown in Figure 3, this process includes two steps. First, a diamond tip was used to make a zigzag trace scan on PC sample surface to form bundles based on friction-induced theory. This process was called first-scan. The scratching angle in first-scan were set to 90° and 0° to form the first-scan bundles. Then, the scratching angle in a second scan angle were set to 0°and 45° to form the second-scan bundles. The bundles formed during the two-scan process will be overlapped to achieve nanodot arrays. By changing the relative angle between the first and second-scan, different oriented bundles can be easily achieved. Moreover, by controlling the feed between two scratching trajectories, various dimensions of nanodots can be achieved easily. In conclusion, this method based on friction-induced bundle overlapping provides a simple, relatively low cost and feasible method to fabricate nanodots arrays on polymer materials. But there is also a shortcoming exists in the above method, such as the poor uniform in the dimension of nanodots and low density of nanodots arrays, which may have a negative effect on applications of nano-optics and SERS measurement. In order to improve the density of the nanodot arrays, He et al. [81] proposed a dynamic plowing lithography (DPL) method to fabricate nanodot arrays on PMMA thin films by overlapping of two machined nanogrooves as shown in Figure 4. First, a nanogrooves array with a given separation distance is fabricated. Second, another nanogrooves array is scratched on the same area of the first-scratch. In this process, the scratching angle must be changed so that the two nanogrooves arrays can be overlapped to form nanodot arrays. Finally, a third scratch can also be conducted so as to form higher-density nanodots arrays. Checkerboard nanodots arrays with a density of 1.3 × 10^9^ dots/mm^2^ and diamond-shaped nanodots with density of 9.6 × 10^8^ dots/mm^2^ have been obtained by a two-step DPL method. In addition, a three-step DPL method is utilized to fabricate hexagonal nanodots with a density of 1.9 × 10^9^ dots/mm^2^. This indicates that this method based on the DPL technique provide a possibility for fabricating higher density of nanodot arrays on polymer materials, which has the potential in preparation of nano-optics sensors and SERS substrate.

**Figure 5:**
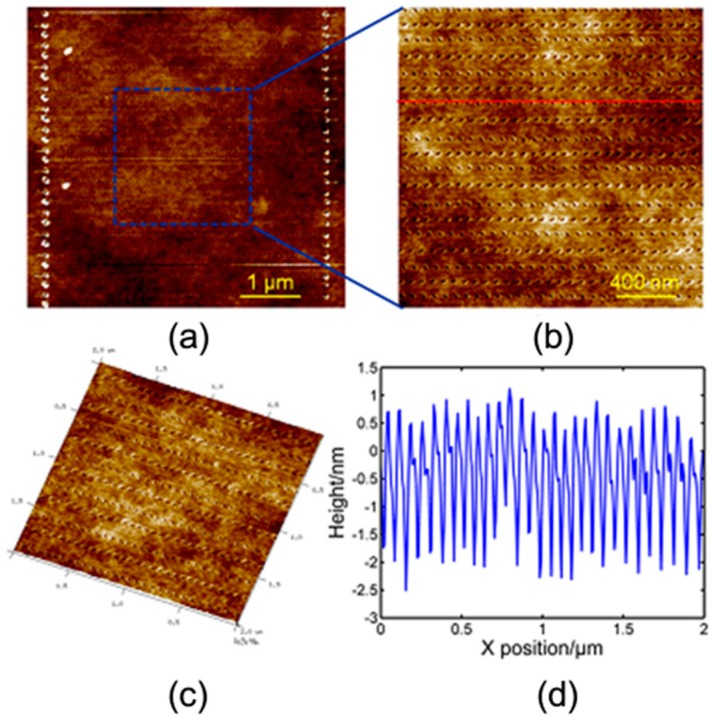
Surface morphology images of nano-pit arrays at a scanning speed of 400 μm/s in the scan size of 5 μm measured by AFM: (**a**) 2D images, (**b**) zoom-in image of figure (**a**) at a dimension of 2 μm, (**c**) 3D image of (**b**), (**d**) cross-section of (**b**) [82].

As for fabrication of nanopits, the approach widely used now is using a tip to indent into the sample with a relatively large normal load so as to achieve an ordered nanopit with given depth. In this method, the dimension of the nanopit is determined by the applied normal force, the geometry of the tip and the properties of the polymer material. In particular, the depth of the nanopit is smaller than that predicted by the theoretical model due to the elastic recovery of the material after scratching. Many scholars have obtained nanopits using the TBN method and studied the influence of scratching parameters on achieved nanopits. He et al. [82] used the DPL method, which is based on the tapping mode of AFM to scratch nanopits on PMMA thin films with high efficiency. In this study, a critical scratching velocity was observed to form nanopit. Only when the scratching velocity larger than 100 μm/s, can nanopit be achieved, or a nanogroove be scratched. This method provides an approach to scratch nanopits with a high throughput of 4800–5800 pits per second, as shown in Figure 5. Moreover, in the following study of He et al. [83], they found that the mean molecular weight and driving amplitude almost had no effect on nanopits scratched on PMMA thin film. Three kinds of polymer film including PMMA, PC and polystyrene (PS) were used to study the influence of material on the critical value of scratching velocity for the transformation from nanogroove to nanopit. And the result showed that the critical velocity of PC and PS is about 20–30 μm/s, which is much smaller than that of PMMA due to the different elastic moduli of three kinds of thin film. This study gives out a relatively accurate critical velocity value of scratching nanopits in PMMA, PS and PC thin films, which makes data storage on these films possible.

### 3.2. Fabrication of Nanogroove/Channel

The polymer materials are widely used in the biological detection owing to its good biocompatibility and can be used in the preparation of nanofluidic chips. The nanogroove is a key component in nanofluidic chips, known as nanochannel, the dimension of which determines the application of the nanofluidic chips directly. Therefore, in order to meet the requirement in the field of nanofluidics, many scholars have proposed several approaches to obtain nanochannel structures. Scratching on polymer materials exhibits an advantage of nanoscale machining accuracy using the TBN method. Thus, fabricating on polymer materials using the TBN method is becoming a hot research topic. The existing nanogroove scratching methods by the TBN approach can be divided into three kinds of approach according to different fabrication principles, including single-pass scratching, multi-pass scratching and nanomilling.

#### 3.2.1. Single/Multi-Pass Scratching Approach

**Figure 6:**
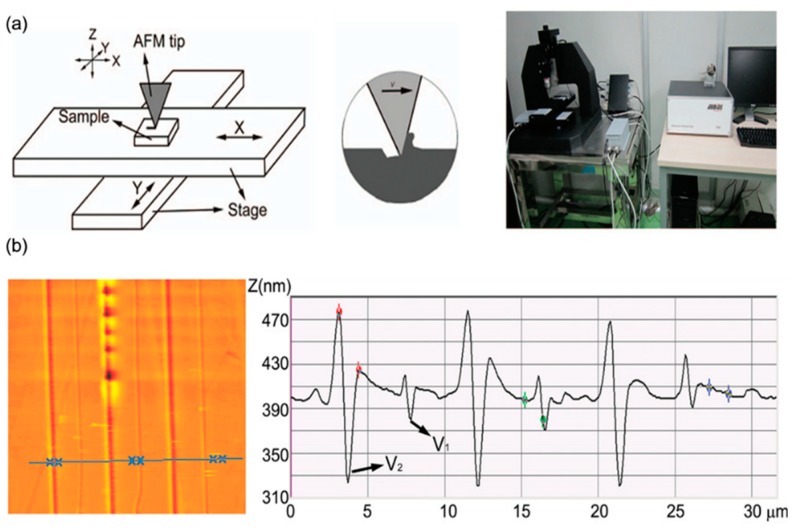
Schematic diagram of the formation process of nanodot arrays: (**a**) schematic of the modified AFM system with a large-scale rang of X-Y stage. (**b**) the morphology and cross-section image of typical nanoline arrays scratched by the above system [79].

First, for single-pass scratching approach, the tip scratches on sample with a given normal load only once. Therefore, the aspect ratio of nanogroove is determined by the geometry of the tip, and the depth depends on the applied normal force. Meanwhile, the maximum length of the nanogroove is determined by the maximum moving range of the AFM stage. Thus, a larger depth can only be achieved by increasing the applied normal load in single-pass scratching approach. Although this method provides a simple and easy approach to achieve a desired nanogroove, lots of shortcomings still exist resulting in the application of the nanogroove becoming a challenge. First, the range of the X-Y stage for a commercial AFM system is limited by several tens of micrometers. In order to solve this problem, Hu et al. [79] used a large-scale high-precision stage to replace the original stage in the commercial AFM system, as shown in Figure 6. The maximum moving range of the X-Y stage is 100 mm × 100 mm. Using this modified AFM system, the nanochannel with a length of several hundred micrometers or even millimeter scale can be easily fabricated by the TBN method. Nanogrooves arrays with dimension of 1 mm × 0.5 mm was fabricated by the TBN method using this system. Another problem is the depth of the nanogroove is determined by the applied normal force, so a larger depth can only be achieved by a larger normal load. Under this condition, extreme tip wear will be observed. Thus, how to obtain a nanogroove with a large machined depth and less tip wear is another limitation for the TBN method. In addition, the dimensions of nanogrooves are limited by the size of the tip and the normal load, thus, it is difficult to achieve a nanogroove with an arbitrary aspect ratio using the TBN method. To rise above the limitation, Geng et al. [84] compared the nanogrooves machined on the PMMA thin film with different thicknesses by single-pass, multi-pass and vibration-assisted scratching method. The multi-pass scratching method was used to enlarge the size of the machined nanogroove, but an extreme tip wear was observed during the scratching process. Considering tip wear, the multi-pass method is not the best way to scratch a wider and deeper nanogroove compared to the vibration-assisted single-pass scratching method. For results obtained by Geng et al. [84], the vibration-assisted method has been proved as a feasible approach to achieve a deeper and wider nanogroove rising the limitation from the value normal load applied by the tip. Thus, this method exhibits an outstanding capacity in reducing the tip wear occurring in the machining process. 

#### 3.2.2. Nanomilling Method

**Figure 7:**
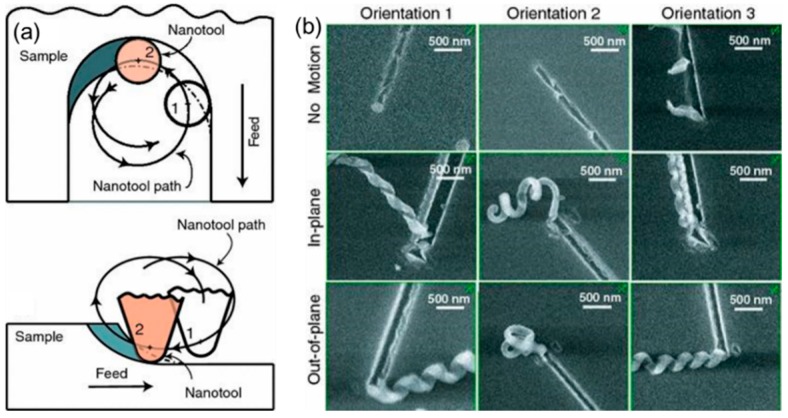
The schematic diagram of nanomilling: (**a**) the trajectory of the tip when scratching sample, (**b**) the scanning electron micrographs (SEMs) of nanochannels fabricated by nanomilling [85].

Nanomilling was known as a rotation tip-based nanoscratching method, which is first proposed by Gozen et al. [85]. During nanomilling process, the tip is regarded as a small cutting tool and conducts a rotary motion just like that in traditional milling process in macroscale, as shown in Figure 7a. Gozen et al. [85] performed nanomilling fabrication on a PMMA bulk sample. A modified AFM system using three piezoelectric actuators to control the rotary motion of the tip was built up. Based on the established nanomilling system, desired nanochannels with long curled chips were fabricated, and the scanning electron microscope (SEM) images of the machined nanochannels were shown in Figure 7b. Compared with the traditional TBN method, nanomilling exhibits an outstanding feasibility in machining the nanochannel with arbitrary aspect ratio, improving the accuracy of the fabricated nanostructure and the materials removal state is mainly in cutting mode, which shows huge potential in fabricating complex nanostructure with good machining quality in a controllable way. However, something defective also exists in that the fabrication efficiency needs to be improved furthermore when scratching more complex structures, and the tip wear during the scratching also cannot be neglected. A similar work was conducted by Wang et al. [54], when the original stage of commercial AFM system was replaced by a piezoelectric actuator to generate rotational relative motion between the tip and the sample. In this work, nanochannels with desired dimension were obtained in an easy and controllable way, which can meet the demand of preparation of nanofluidic chips.

**Figure 8:**
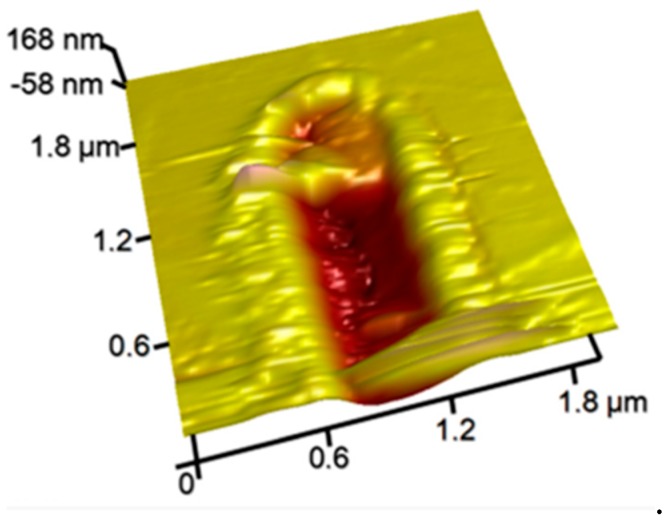
Nanochannel fabricated by two-step nanomilling approach [86].

**Figure 9:**
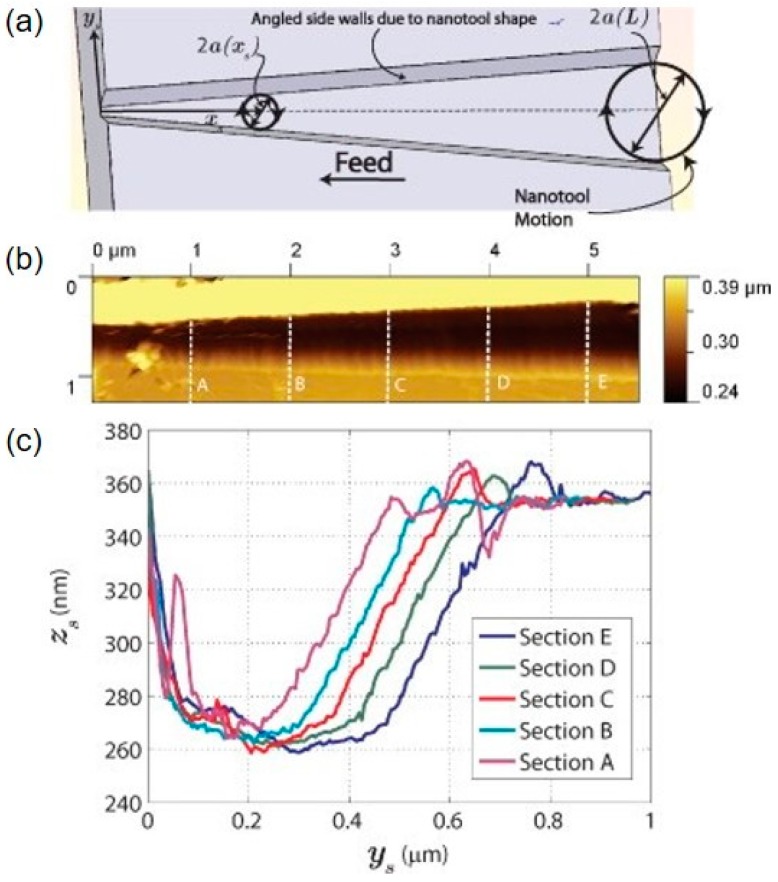
Nanochannel with variable width fabricated by nanomilling approach: (**a**) the schematic diagram of variable width fabrication method, (**b**) the AFM image of nanochannel, (**c**) the cross-section views of section A, B, C, D and E [87].

Li et al. [86] also used the nanomilling approach to scratch on a PMMA bulk sample. In their work, they focused on how to reduce the pile-up accumulated on both sides of nanochannels so as to be more suitable for the preparation of the nanofluidic chip. A two-step scratch was observed to enlarge the depth of the nanochannel. The outer profile of nanostructure was fabricated during the first half cycle process and the inner profile was obtained in the second half-cycle scratching process. As shown in Figure 8, the polymer material was removed as chips but not pile-up, and the formed chips could be cleaned up using a tip to scan on the scratch-region in the contact mode. Therefore, nanochannels with small pile-up on both sides were obtained easily. This indicates that this method shows a huge potential ability in reducing the height of the pile-up accumulating on both sides of the nanochannel compared to the single-pass scratching method. Besides, nanochannel with variable widths was fabricated using nanomilling method on PMMA bulk sample by controlling the rotation radius of the tip [87]. The amplitude of the rotation of the tip varies from 0 to 320 nm during the scratching process, and the machined result is shown in Figure 9. 

**Figure 10:**
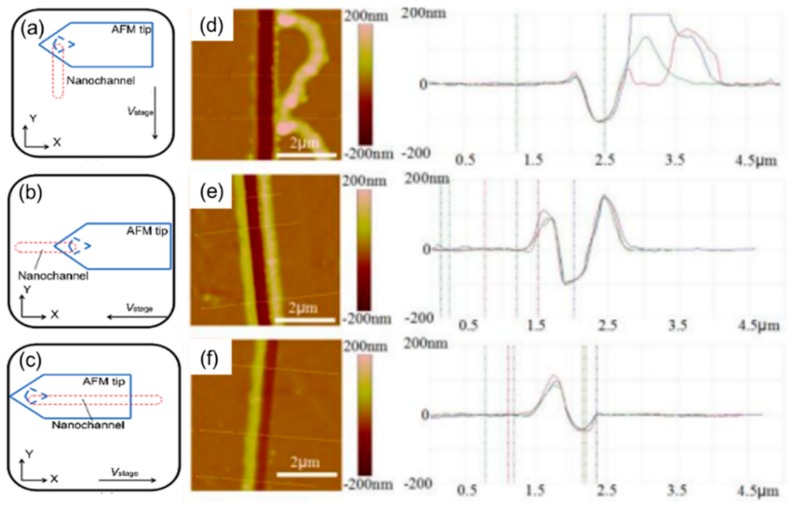
The influence of three feed directions on the formation of pile-up fabricating on PMMA bulk sample: (**a**) along positive direction of *y*-axis, (**b**) along positive direction of *x*-axis, (**c**) along negative direction of *x*-axis, the opposite direction of (**b**), (**d**), (**e**), (**f**) the AFM image of nanochannels fabrication along the directions of (**a**), (**b**) and (**c**) [88].

Following the study of Gozen [85], in order to satisfy the demand of achieving nanochannels with no pile-up so as to be applied in the preparation of fluidic chips, Geng et al. [88] studied the influence of feed direction on the formation of the pile-up on both sides of nanochannel. Three typical feed directions were chosen to fabricate nanochannels on PMMA bulk sample by nanomilling approach under the same machining parameters. As shown in Figure 10, chips could only be observed when scratching with the feed along the positive direction of the *y*-axis, while, using the other two feed directions, pile-up formed on both sides of the nanochannel. Moreover, the chips can be cleaned up by scanning the scratch-region using a tip in the contact mode. Therefore, only the nanochannels obtained with the feed along the positive direction of *y*-axis were fit for preparing the nanofluidic chips. 

**Figure 11:**
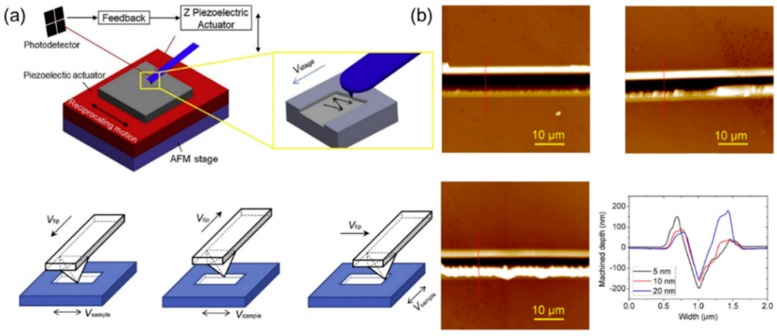
(**a**) The schematic diagram of fabricating nanogrooves using the combination of the TBN method and reciprocating motion, (**b**) nanogrooves obtained by the approach of (**a**) [89].

In order to improve the scratching efficiency of the nanomilling process, Geng et al. [89] built up a modified AFM system based on a piezoelectric actuator to improve the scratching velocity, which can also be used to control the width of the scratched groove, the schematic diagram is shown in Figure 11. In this method, the piezoelectric actuator was used to control the motion of the sample. A scratching velocity of 5 m/min could be achieved when a frequency of 40 kHz was provided by the actuator, which is 5 times as much as that of the work conducted by Gozen [85]. This approach exhibits an ability to improve the throughput of scratching the PMMA sample by the TBN method, which could be considered as a potential approach used in the area of producing NEMS devices. In their study, the influence of three typical feed directions, scratching velocity and frequency of actuator on the depth of achieved groove and the formation of the chip were also studied in detail. But there is an unknown issue in that the limitation of cutting speed can be achieved by this proposed method is not clear enough and further study needs to be conducted.

**Figure 12:**
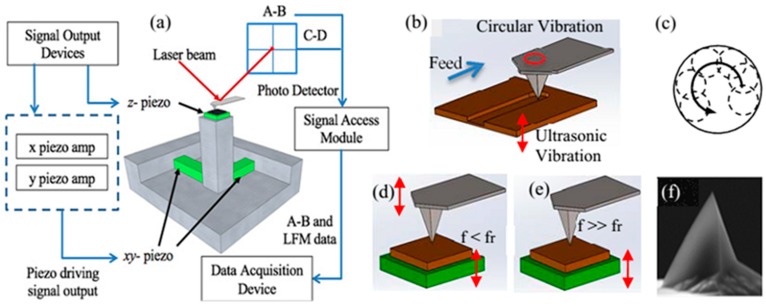
The schematic diagram of the vibration-assisted method: (**a**) modified AFM system, (**b**) the diagram of machining, (**c**) the circular path of the tip, (**d**) tip vibrates without ultrasonic, (**e**) tip vibrates with ultrasonic, (**f**) the SEM image of the tip used in this method [90].

Besides the machining efficiency, the tip wear is also a crucial issue in the nanomilling process. Further improving on reducing the wear of the tip is still needed to be studied. Zhang et al. [52,55,90] first introduced the ultrasonic frequency vibration into nanoscale fabrication based on the TBN method. As shown in Figure 12, the scholars use three piezoelectric actuators to control the relative motion between the tip and the PMMA thin-film sample. A relatively high ultrasonic frequency with 2 MHz is applied to the piezoelectric actuator in the *z*-axis direction to generate an ultrasonic force between the tip and the sample surface to enlarge the penetration depth of the tip. In addition, a frequency circular vibration with 10 kHz is applied to the XY-plane stage to regulate the width of the nanostructure and increase the speed of fabrication so as to improve the nanoscratching efficiency. Compared with the traditional nanomilling process, the combination of nanomilling and an ultrasonic vibration-assisted approach can obtain a wider and deeper nanogroove under an order of nanonewton normal load, which is much smaller than that of commonly used in the nanomilling process with several tens of micronewtons. The tip wear can be reduced accordingly. In this method, nanogrooves with various dimensions ranging from several tens to hundreds of nanometers can be achieved easily, and the speed of fabrication can reach tens of microns per second. Moreover, the combination of tip and cantilever is regarded as a weak stiffness system, which is similar to the traditional macroscopic process of ultrasonic vibration-assisted machining [91]. 

### 3.3. Fabrication of 3D Nanostructure

Although the TBN method has been used for nanomachining nanostructures for many years, the fabrication of 3D nanostructure in nanoscale still faces great challenges, especially a complex 3D nanostructure [61,92]. The nanomachining mechanism based on the TBN method can be summarized to two main ways, including the remove of the material and the deformation of the material. The former is suitable for metallic and semiconductor materials, which can generate chips during the nanoscratching process. However, the latter is mainly fit for polymer materials owing to its high viscoelasticity. Therefore, without formation of any cutting chips, the polymer materials are squeezed to deformation by the tip to form the 3D nanostructure, which is called the ploughing process. A typical 3D nanostructure is well known as bundles, which was first observed by Leung et al. on the surface of PS in 1992 [93], exhibiting huge potential in the area of optical phase grating, diffraction grating of spectrometer and other optical elements. It is a friction-induced periodic nanopattern that forms perpendicular to the scanning direction when using a tip with a stiff cantilever to scratch on polymer materials. The formation mechanism of bundles is still unknown clearly. Other 3D nanostructures like stair-like pattern [49], logo [2], facial profile [2] and so on have been achieved in the past few years, but have not been promoted to the application fields. 

**Figure 13:**
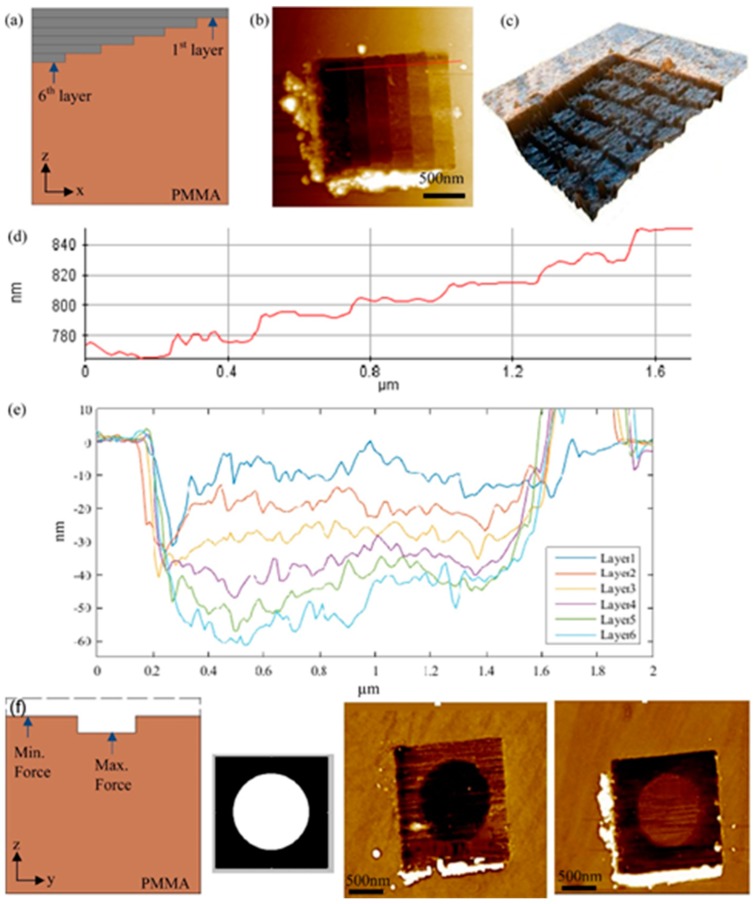
Stair-like 3D nanostructure, a concave cycle in a square and a convex cycle in a square were fabricated by ultrasonic vibration-assisted approach based on the TBN method: (**a**) schematic of layer-by-layer based 3D fabrication process of 6 layers nanostructure, (**b**) atomic force microscope (AFM) image of 6 layers nanostructure, (**c**) 6 layers nanostructure in a 3D view, (**d**) cross sectional profile, (**e**) cross section view of every layer, (**f**) bitmap (BMP) image of a concave circle in a square and the AFM images of concave circle in a square and convex circle in a square [49].

In order to reduce the tip wear during the scratching process and enlarge the size of the machined nanostructures, other energies including ultrasonic and thermal energy were integrated with the TBN method to fabricate 3D nanostructures. Zhang et al. [52] first introduced the ultrasonic vibration-assisted approach into the TBN method and have achieved various complex 3D nanostructures ranging from the logo of the lab, the figure of Steve Jobs to stair-like patterns. After that, some studies have been conducted by Deng [49], which follow the work of Zhang et al. in 2012 [52]. In their studies, two nanoscratching methods are used to fabricate 3D nanostructures, including removing material in a layer-by-layer strategy and fabricating according to every scratching path with a pre-set normal load. The ultrasonic vibration-assisted nanoscratching experiments are conducted on a PMMA thin film, as shown in Figure 13, a 6 layers stair-like nanostructure was obtained by using the layer-by-layer nanoscratching method and it only took a few minutes to machine the nanostructure owing to a high frequency of the XY-plane vibration stage. As shown in Figure 13, a concave cycle in a square and a convex cycle in a square are also fabricated on the PMMA film by the nanoscratching method, and each scratching pass with a given normal load relies on the pre-set grey-scale image. These two nanostructures can also be achieved in a few minutes, which indicates that the vibration-assisted method is a high-efficiency approach. 

**Figure 14:**
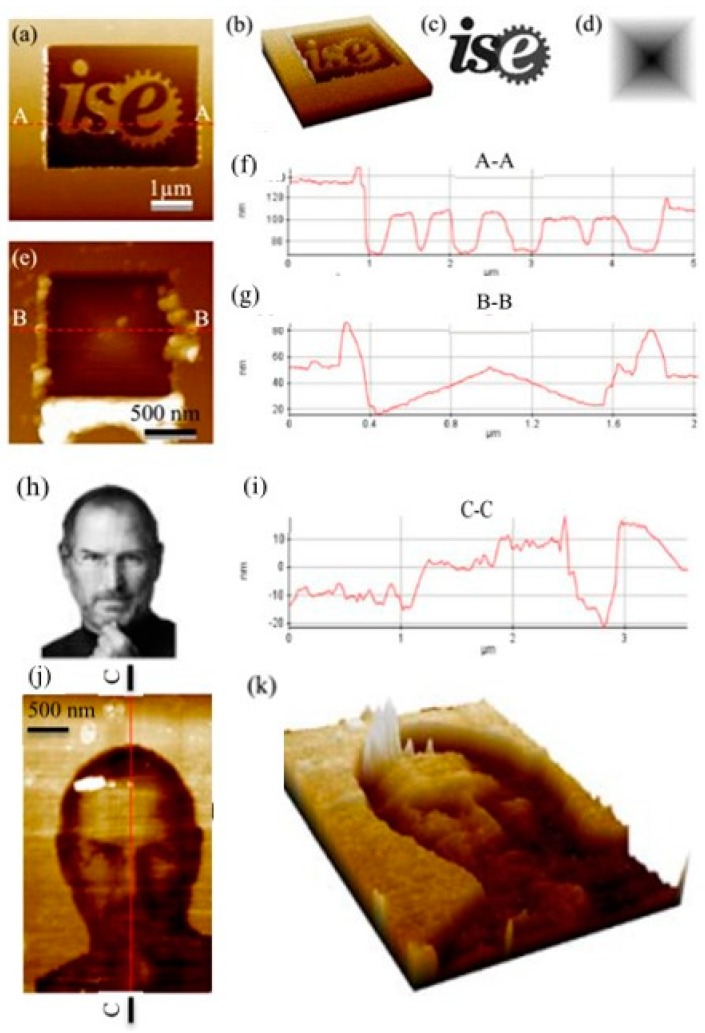
“ise” logo, pyramid 3D structure and the profile of Steve Jobs were fabricated by the ultrasonic vibration-assisted approach based on the TBN method: (**a**) 3D nanostructure of letters “ise”, (**b**) 3D view of “ise”, (c) bitmap images of “ise”, (**d**) bitmap images of pyramid, (**e**) fabricated pyramid nanostructure, (**f**) the image of “A-A” cross section, (**g**) the image of “B-B” cross section, (**h**) bitmap images of Jobs’ profile, (**i**) the image of “C–C” cross section, (**j**) 3D nanostructure of Job’s profile and (**k**) 3D view of Jobs’ profile [90].

Moreover, more complex 3D nanostructures like logo of a lab and facial image were fabricated by Deng et al. [90]. As shown in Figure 14, using an ultrasonic vibration-assisted system similar to previous used [49], 3D nanostructure of letters “ise”, “NSF” logo and 3D view of Jobs′ profile were successfully fabricated on a PMMA thin film by a constant-height mode of the AFM system, which is the main difference compared to previous studies. The operation can be achieved by controlling the absolute height of Z-scanner of AFM. Using this method, desired complex nanostructures with high-precision can be easily obtained by regulating the relative position between the tip and the sample surface. 

The above studies focused on how to improve the efficiency and precision of the nanostructures based on the vibration-assisted method, but few scholars pay attention to the processing mechanism by the vibration-assisted method. Kong et al. [51] established a dynamic nanofabrication force model to predict the nanoscratching force during the fabrication process so as to obtain a high-precision nanostructure and improve the efficiency of machining. In this model, the engagement between the tip and the sample instantaneously was taken into account in each single rotation cycle of the tip. As a result, an experimental dynamic fabrication force model was established based on a discrete voxel method, and the material remove rate was expressed as a function of nanoscratching parameters. Nanoscratching experiments have been performed on a PMMA thin film to verify the prediction model of the dynamic nanoscratching force. Results showed that the experimental machining force agrees well with the theoretical force. This work first gives a theoretical model to predict the dynamic nanoscratching force at every moment when using the vibration-assisted method, which provides a possibility to calculate the machining force in advance so as to control the preset parameters used in scratching and obtain nanostructures with higher precision. 

**Figure 15:**
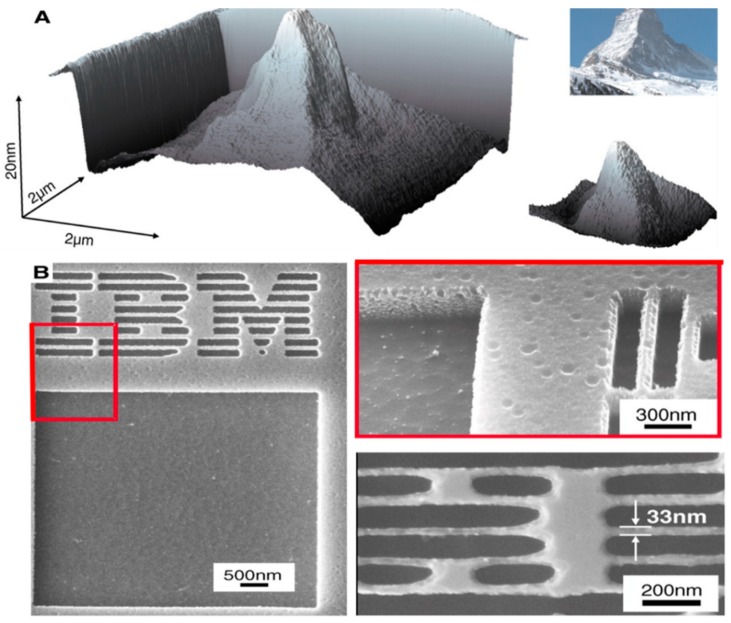
3D structures fabricated by thermal tip: (**A**) 3D sculpture of the Matterhorn. (**B**) 3D image of the IBM letters [94].

In addition, thermal energy was also integrated with TBN method due to the heat-sensitivity of the polymer materials. As shown in Figure 15, a 3D sculpture of the Matterhorn was fabricated using thermal tip based on layer-by layer method on phenolic compound resist by controlling the combination of heat and force. As well, the letters of IBM were fabricated for the purpose of display rather than application by David et al. [94]. This method exhibits huge potential in fabricating 3D nanostructures, especially on high heat-sensitivity polymer materials. Moreover, this method combines the mechanical processing and thermal-fabrication method together, which is known as thermomechanical coupling machining, and thermal energy plays an important role during the machining process. Therefore, the dimension not only depends on the geometry of the tip but also the applied normal load, thus, higher accuracy can be obtained easily compared to nanostructures fabricated by the tip without thermal energy. Besides, it only takes several microseconds to achieve 3D nanostructures with a dimension of several micrometers, which indicates the higher efficiency of this technique. 

To sum up, although lots of 3D nanostructures have been achieved on the polymer materials using the TBN method, it is still a challenge to machine complex 3D nanostructures and a further study needs to be conducted in the future. Moreover, most 3D nanostructures existing now are only for the purpose of display rather than application. As for applying 3D nanostructures to industrial production, there is still a long way to go.

## 4. Applications 

Some polymer films, such as PMMA, exhibit a potential application in nanopattern transferring techniques owing to good etching resistance. The good machinability of some polymer thin-films leads to lots of scholars considering using the TBN method to create nanopatterns on these films. The existing nanostructures fabricated on polymer materials by the TBN method, including nanodot, nanogroove and 3D structures, show a potential application in the fields of Raman detection, nanofluidics and nanosensors. In this section, the applications of the TBN approach can be divided into three parts. First, based on the top-down material removal process TBN method, the typical applications are nanopatterns transferring to hard substrate, data storage on polymer sample, and preparation of nanofluidic chips. Second, based on the mechanism of adding material after the scratching process, the TBN method is integrated with the lift-off process to fabricate nanowires. Third, other applications based the TBN method are discussed as well. The applications of scratching on polymers by the TBN method are summarized into several points as shown in Table 1.

### 4.1. Applications Based on Material Removal Theory by TBN Method

**Figure 16:**
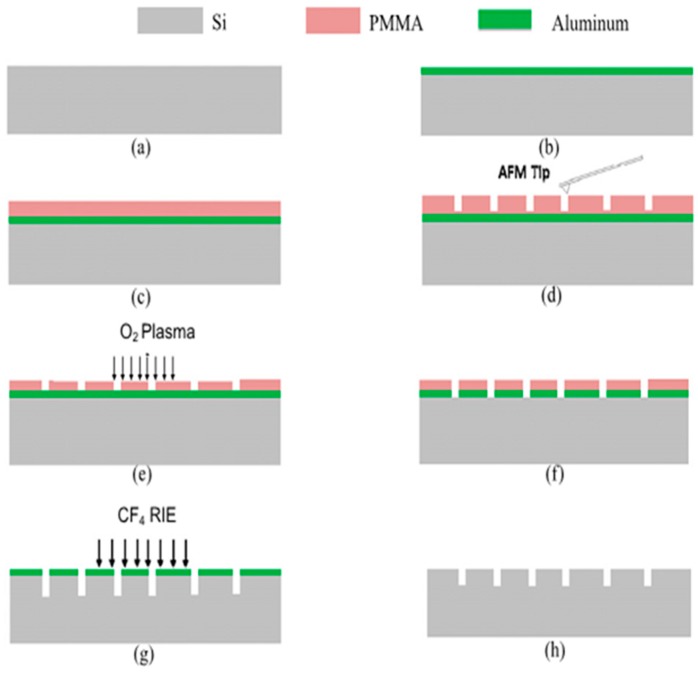
The transfer procedures of nanostructures formed by the TBN method using reactive ion etching (RIE): (**a**) starting from cleaned silicon substrate, (**b**) aluminum deposition on silicon substrate, (**c**) polymethyl methacrylate (PMMA) spin-coating on aluminum layer, (**d**) machining patterns on PMMA, (**e**) PMMA etching in O_2_ plasma till aluminum layer surface is exposed, (**f**) aluminum etching to create mask, (**g**) silicon etching using reactive ion beam etching (RIE), (**h**) aluminum mask stripping and sample surface cleaning [52].

Generally speaking, a classical application of the polymer thin film is employed as a resist to be scratched using the TBN method to obtain nanopatterns on hard materials by following wet or dry etching process, such as silicon and silicon dioxide. Zhang et al. [52] did some work based on the above idea. As shown in Figure 16, the process can be mainly divided into eight steps. First, the silicon substrate was cleaned in acetone and alcohol to keep clean. Then, a layer of aluminum (Al) was deposited onto silicon substrate by a thermal evaporator and later a layer of PMMA film was spin-coated on the Al layer. In the fourth step, desired nanopatterns were fabricated by the TBN method on the PMMA thin film, which was then etched in O_2_ plasma till the Al layer was exposed. In the sixth step, the Al layer was etched to generate a mask for the etching of silicon in the following process. Finally, the reactive ion etching (RIE) method, a dry-etching process, was used to etch silicon in the atmosphere of CF_4_ and the desired nanopatterns can be transferred to the silicon substrate successfully. This method represents the most common application of fabrication on the polymer materials by the TBN method and has been widely used during the past few years. 

**Figure 17:**
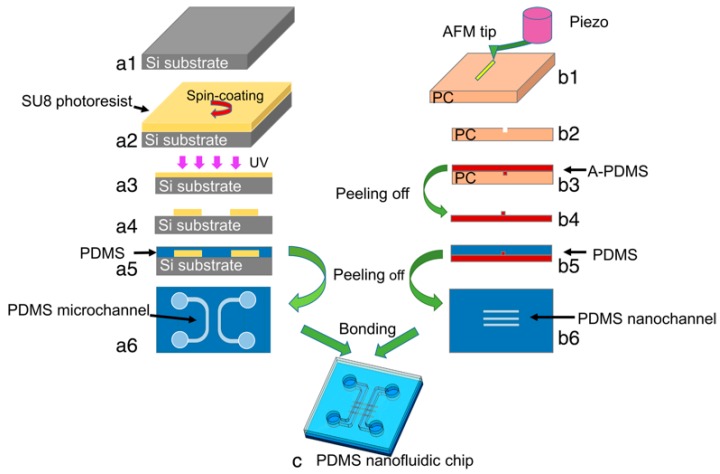
The preparation procedures of nanofluidic chip based on the TBN method: (**a1**)–(**a6**) working steps of microchannel fabrication on a polydimethylsiloxane (PDMS) chip, (**a1**) silicon sheet used for lithography substrate, (**a2**) spin-coating of SU8 photoresist on Si substrate, (**a3**) exposure of the SU8 layer to ultraviolet (UV) light, (**a4**) obtained convex microstructures, (**a5**) PDMS coating on microchannel mould, (**a6**) final PDMS chip with microchannels, (**b1**)–(**b2**) working steps of nanochannel fabrication on a PDMS chip, (**b1**) AFM tip scratches on polycarbonate (PC) sheet, (**b2**) obtained nanochannel mould after scratching, (**b3**) A-PDMS coating on nanochannel mould, (**b4**) A-PDMS chip with convex nanostructures, (**b5**) regular PDMS coating on A-PDMS mould, (**b6**) final PDMS chip with nanochannels, (**c**) PDMS nanofluidic chip after bonding [54].

Another application of the nanogrooves fabricated by TBN approach is preparation of nanofluidic chips. Nanochannel, that is, nanogroove on the nanofluidic chips, is a key component, which is usually utilized to conduct biological detection. Wang et al. [54] first proposed a fabrication method for nanochannels used in nanofluidic chips by the TBN method, and the details of the preparation procedures are shown in Figure 17. They used a piezoelectric actuator on the X-Y plane to generate a vibration of the sample and the relative motion between the tip and the sample is like the traditional milling process, hence, this method is named nanomilling. As shown in Figure 17, the formation of the nanofluidic chip can be divided into five main steps. The first step is scratching nanogrooves on PC bulk sample to generate desired nanochannels. The nanochannels on the PC surface can be used as a mould for the following procedures. Then, a layer of polydimethylsiloxane (PDMS) was coated on the PC sample with nanochannels and the layer of PDMS was peeled off after baking on a hot oven at 80 °C for 4 h. A PDMS convex mould of the nanochannel called A-PDMS, was achieved shown as Figure 17b3, the process of which is known as the first-step transfer. In the third step, another layer of PDMS was coated on A-PDMS, which was peeled off later to form a concave mould, as shown in Figure 17b6. Finally, the nanochannels were transferred successfully from the PS bulk sample to PDMS. As a result, the nanochannels scratched by TBN method and the microchannels generated by lithography were bonded together to form a PDMS nanofluidic chip, which was then used to verify the change of electric current in microchannels and nanochannels using KCl solution with a concentration of 1 mM. This work indicates that the nanochannels used in nanofluidic chips can be fabricated easily by the TBN method compared to the traditional lithography method. However, a shortcoming may exist that the material accumulation on both sides of the nanogroove may be squeezed into the nanochannel during the bonding process and may have a negative effect on the flow of liquid in the nanochannels. 

**Figure 18:**
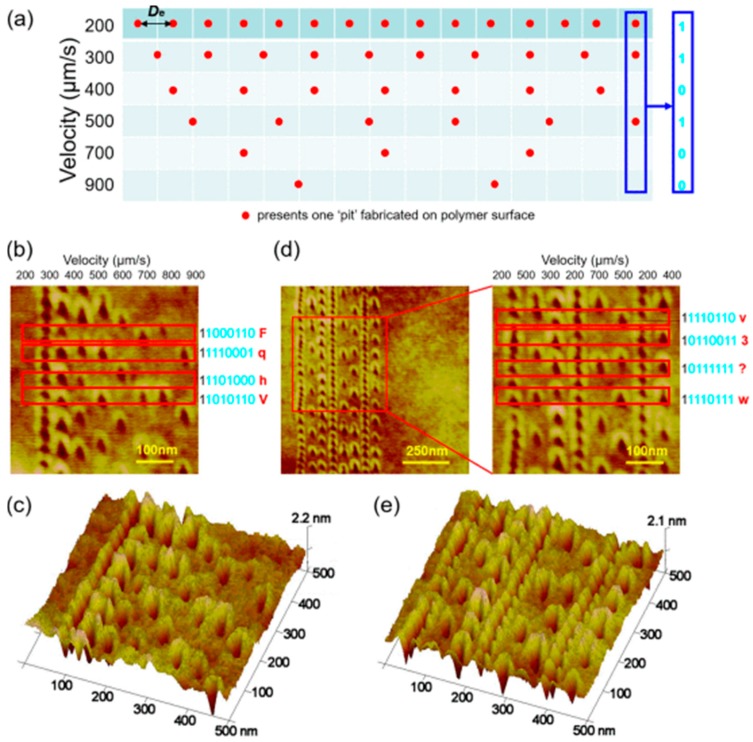
Creating 8-bit American Standard Code for Information Interchange (ASCII) codes by DPL method scratching on PMMA thin-film using different velocities: (**a**) schematic of the nanoscale pits fabrication with various scratching velocity, (**b**), (**c**), (**d**) and (**e**) morphologies and 3D images of fabrication results for arrays of pits on polymethyl methacrylate (PMMA) film surface [83].

In addition, another application proposed by He et al. [83] recently has the potential to be used in data storage. As shown in Figure 18, several nanopits arrays were fabricated by DPL method on the surface of the PMMA thin films with various scratching speeds. It can be observed from Figure 18b that the scratching velocities were set to 200–900 μm/s, the interval was set to 100 μm/s and eight nanopits arrays with different distances between two adjacent nanopits can be obtained with the different scratching velocities. These were concave pattern symbols “1” and the flat surface without nanopit symbols “0”, therefore, 8-bit American Standard Code for Information Interchange (ASCII ) codes of “F, q, h, V” were obtained and those of “v, 3, ?, w ” were also obtained in Figure 18d with the scratching speeds of 200, 500, 300, 200, 700, 500, 200, 400 μm/s, respectively. This method provides a simple approach to high-density storage of data on surface of the polymer materials.

### 4.2. Applications Based on Adding Material Theory by TBN Method

**Figure 19:**
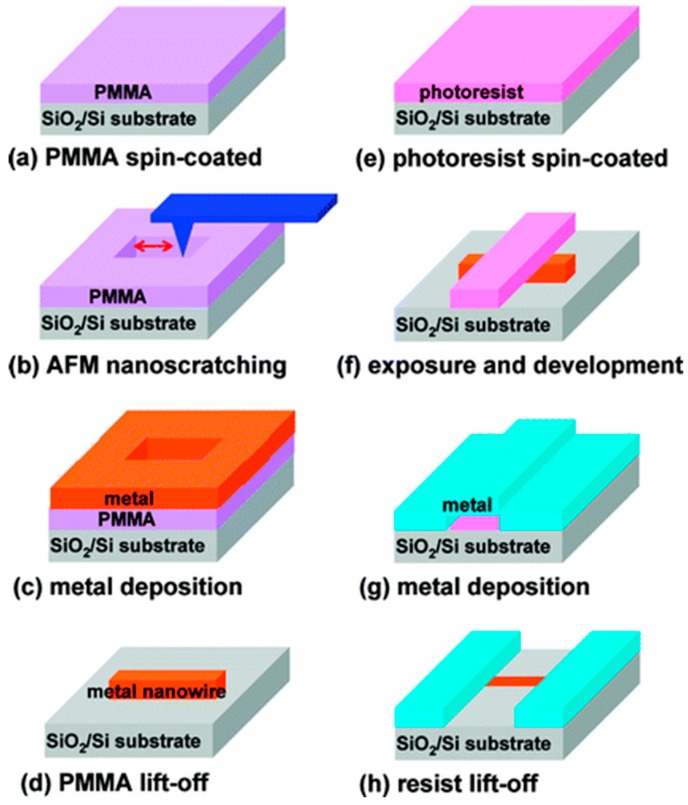
The fabrication procedures of single Au nanowire using the combination of the TBN approach, lift-off and the photolithography method: (**a**) polymethyl methacrylate (PMMA) was spin-coated on the silicon/silicon dioxide substrate, (**b**) nanogroove was fabricated on the surface of PMMA thin-film by the tip-based nanomachining/nanoscratching (TBN) method, (**c**) a layer of metal was deposited above the PMMA thin-film, (**d**) metal nanowire was fabricated by lift-off process, (**e**) a layer of photoresist was spin-coated on the silicon/silicon dioxide substrate with metal nanowire, (**f**) the photoresist was exposed and developed, (**g**) a layer of metal was deposited above the substrate with metal nanowire and photoresist, (**h**) the resist was removed by lift-off process [53].

**Figure 20:**
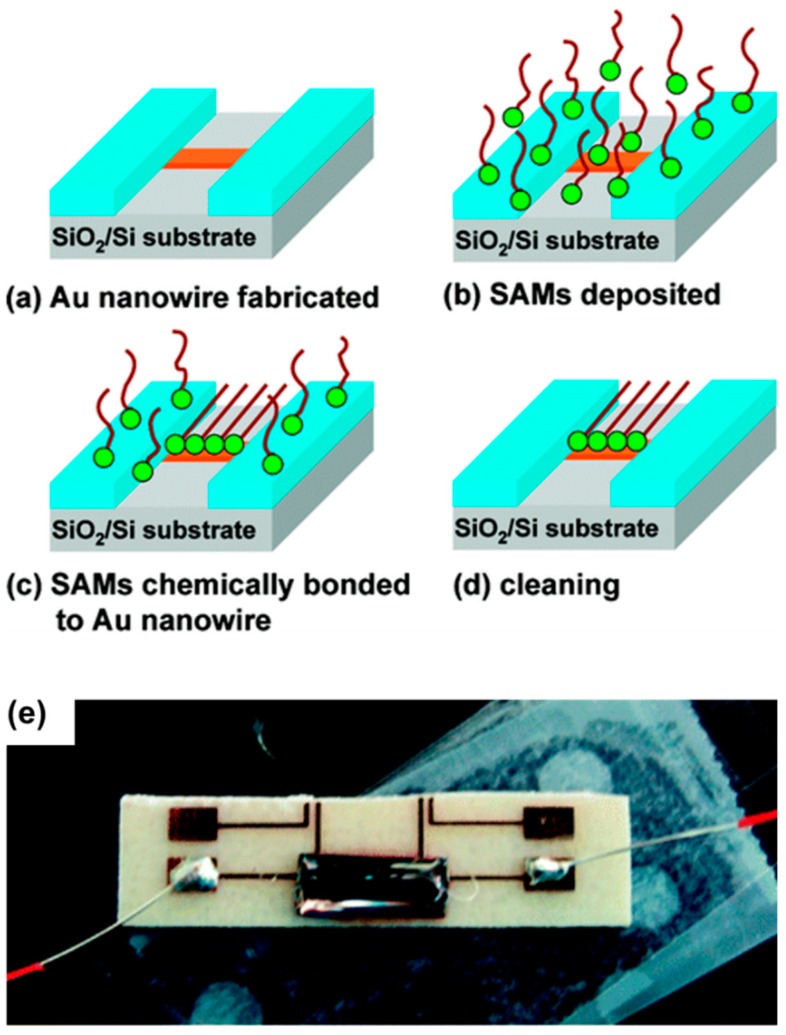
The process of detecting the concentration of octadecanethiol molecular solution using a single Au nanowire: (**a**) Au nanowire was fabricated on the silicon/silicon dioxide substrate, (**b**) self-assembled monolayers (SAMs) were deposited on the silicon/silicon dioxide substrate with Au nanowire, (**c**) SAMs chemically bonded to Au nanowire, (**d**) other SAMs not bonding to the Au nanowire was cleaned and (**e**) top-view photograph of a resistance measurement unit using Au nanowire [53].

For adding material after scratching process, a typical application is using TBN method combining with the lift-off technique to generate nanowire, which was first proposed by Yu-Ju Chen et al. [78,95] in 2005 and nanowire with a minimum width of 50 nm was obtained successfully in their work and the process mainly includes three steps. The first step is spin-coating a PMMA thin film resist with a given thickness onto silicon dioxide and using a tip to cut through the film with a relatively large normal load in order to generate ordered nanogrooves. Second, a layer of metallic material, such as Au, Al and Cu, was deposited onto the PMMA film resist by e-beam evaporation. Finally, the sample was soaked in acetone solution to remove the PMMA thin-film resist, that is, the lift-off process, then, a nanowire could be obtained. As a result, nanowire arrays were achieved using the combination of TBN method and lift-off process, and the width of the nanowire is about 70 nm. In this method, the dimension of nanowire is determined by that of the nanogroove obtained using TBN method, therefore, the size of the nanowire is not controllable. However, the advantage is that various kinds of metal nanowire ranging from Au, Ti to Al can be achieved using this method in a simple and easy way. The nanowire plays an increasingly important part in nanosensor with the rapid development of NEMS. In the following study conducted by Lin et al. [78], this method has been demonstrated as a new approach to replace (EBL) in resolution and efficiency. Based on the above study, a novel method, which combined several techniques including the TBN approach, lift-off and traditional photolithography, was proposed to fabricate nanowire with electrodes [53]. As shown in Figure 19, the procedures of fabricating single Au nanowire were mainly divided into eight steps. Moreover, using this method, a single nanowire with a thickness of 20 nm was achieved. In particular, the main difference between the nanowire obtained by this method and that in the study of 2005 is that two Ti electrodes were formed on both sides of the Au nanowire, and it could be utilized to conduct chemical sensing. The measurements were performed on two different molecules, that are octadecanethiol (CH_3_(CH_2_)_17_SH) and dodecanethiol (CH_3_(CH_2_)_11_-SH, DDT). The schematic diagram of the measurement process is shown in Figure 20, the mercaptan molecular solution with given concentration was deposited on the nanowire and the concentration of the solution can be obtained by measuring the change in resistance of nanowire before and after depositing the solution. Results showed that the resistance of nanowire increased by about 9% when either kind of octadecanethiol was deposited to cover the nanowire with a thickness of 20 nm completely. This method provides a simple method to detect the concentration of the chemical solution and more applications of nanowire will be published in the days to come.

**Figure 21:**
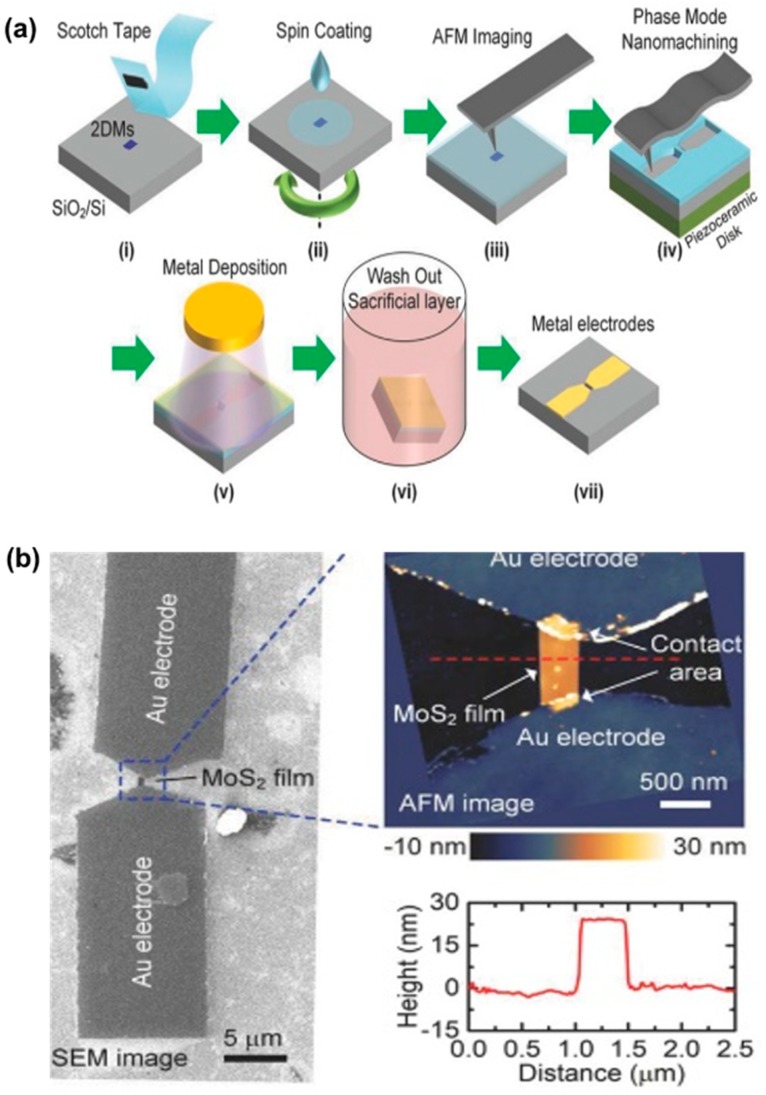
The fabrication of MoS_2_ thin-film transistor based on lift-off and TBN method: (**a**) the detailed procedures of preparing transistor, (**b**) the SEM image of the transisitor and cross-section of the MoS_2_ thin-film transistor [96].

Moreover, a similar study was conducted by Liu et al. [96] recently, whereby the lift-off process was integrated with TBN method to fabricate MoS_2_ thin-film transistor. As shown in Figure 21a, a multilayer of MoS_2_ thin film from crystals of molybdenite by repeating peeling was first transferred to silicon dioxide substrate and then a layer of PS film with a thickness of 40 nm was spin-coated on MoS_2_ thin film. In the third step, two pits with a given shape were scratched on the PS film by the TBN method. Then, a layer of gold with a thickness of 10 nm was deposited on the PS film with two pits. Finally, the sample was bathed in toluene solution then the PS film layer and gold layer on PS film were removed from the sample; this process is known as lift-off. As shown in Figure 21b, a sub-micrometer-dimensional MoS_2_ thin-film transistor with two Au electrodes was obtained. This method makes fabricating 2-dimensional materials (2DMs) devices with sub-micrometer dimension in as easy a fashion as possible, providing an approach to study the size effect on the fabrication of 2DMs devices, meanwhile, which may be widely used in large-scale industrial production in the future. 

### 4.3. Other Applications by the TBN Method

Another novel application has been discovered by Geng et al. [97] by the TBN method scratching on a PC bulk sample, which is used to measure the error motions in the axial and radial direction of a high ultra-precision spindle. When measuring the error motion in the axial direction, the tip was regarded as a small tool to approach the surface of the PC sample with a light normal force so as to prevent the plastic deformation of the sample. Then, the spindle rotated to make a circular motion and the signal change in PZT could be collected; the error motion in the axial direction was then obtained. When measuring the error motion of the spindle in radial direction, a relatively large normal force, in the order of micronewton, was applied to the tip so as to penetrate into the sample and scratch nanogrooves. Then, the spindle rotated to create a 360° circular moving and a circular groove was obtained. By analyzing the depth of the groove and the scratched path, the error motion in the radial direction could be calculated easily. As a result, 124 and 279 nm were measured for the axial error motion and radial error motion, respectively, exhibiting a good fit with those provided by the vendor. This method provides a simple and easy approach in measuring the error motion of an ultra-precision spindle in a reliable way.

## 5. Conclusions

In the past few years, the TBN method has been demonstrated as a powerful approach to fabricate polymer materials with nanoscale resolution and high precision. In the nanoscratching process, AFM is kept in a constant-force mode, and the dimensions of the nanostructures are mainly determined by the tip geometry, tip trajectory and machining properties of the polymer sample. Compared with the fabrication process of metal using the TBN method, the elastic recovery of the polymer materials should be considered, making the establishment of a theoretical model for the machining process the polymer materials more complicated. Therefore, the nanostructures with desired dimensions cannot be achieved easily as the metal materials. In this paper, the current development situation of the TBN method on the polymer materials can be summarized into the following aspects:

(1) The theoretical models of scratching on polymer materials using TBN method were summarized, which take many factors into consideration, including the applied normal load, the scratching velocity, the geometry of the tip, the elastic recovery of the material and so on. During the fabrication of polymers, the elastic recovery cannot be ignored owing to high viscoelastic of polymer materials. 

(2) The status in the fabrication of nanodots/pits, nanogroove/channel, bundles, 3D nanostructures on polymer materials using the TBN method was reviewed. Up to now, nanodot arrays with high density has been successfully fabricated through two main methods by changing the scanning angle in multi-step scratching, and machining nanopits by the DPL approach. As for the fabrication of a nanogroove/channel, three main TBN approaches were used, including single-pass scratching, multi-pass scratching and nanomilling. Bundles, as a special quasi-3D structure, generate perpendicular to the scanning direction when using a tip with stiff cantilever to scan on the surface of polymer sample and the formation mechanism of this is still unknown clearly. Moreover, most 3D nanostructures exist now are only for the purpose of displaying rather than application. As for applying 3D nanostructures to industrial production, there is still a long way to go.

(3) In this review, the applications exist now of scratching on polymer materials by the TBN method and the achieved nanostructures were reviewed, mainly including in the fields of nanosensor, nanofluidic and the transfer of nanopatterns. Among them, the transfer of nanostructures was achieved by the combination of TBN method and other etching techniques, such as RIE, the lift-off process [98], EBL and FIB. 

Although the nanoscale machining technique has been studied for so many years, large-scale industrial production has not been realized yet, the case of which is the similar with the TBN method fabricating on polymer materials. Thus, in order to rise the limitation of itself and achieve industrial application early, more studies still need to be conducted in the future. 

**(1) The establishment of theoretical model.** The existing models now are just suitable for scratching nanogrooves using a given tip by the TBN method, but are not appropriate for scratching other nanostructures, such as nanodot arrays and 3D nanostructures. Therefore, a general model is required to be established fitting for fabricating various nanostructures under different machining conditions by TBN method. Moreover, the formation mechanism of bundles has not been understood clearly up to now. A widely accepted theory is necessary to be studied so as to guide the nanoscratching of bundles on polymer materials by TBN method in a controllable way. 

**(2) Study of the mechanism of tip wear.** In the process of scratching by the TBN method, the tip begins to wear from the moment the tip contacts the sample. The tip wear has a large influence on the machined results, and this point was especially reflected in the scratching process of a nanogroove. The accuracy and uniformity of the groove cannot be guaranteed because of the extremely tip wear, especially under a relatively large applied normal load. Therefore, it is necessary to find a new approach to reduce the tip wear so as to improve the precision of nanostructures as much as possible. But the mechanism of tip wear is still not very clearly understood. Though some scholars have proposed some methods to reduce the tip wear during the scratching process, such as using a diamond tip, it is still short of being used in most experiments due to its high-cost. Moreover, a brand-new method called the ultrasonic vibration-assisted approach is introduced to TBN method to reduce the tip wear, which is a method with high precision and efficiency and is widely used in scratching deeper and wider nanostructures. 

**(3) Advance the application of** the **TBN method.** The existing applications of TBN method scratches on polymer materials ranging from nanosensors, nanofluidics and transfer of nanopatterns, are almost achieved by the combination of the TBN method and other fabrication methods, such as the lift-off process [53,78,99,100,101], lithography and RIE. However, the existing applications remain unused in industrial engineering owing to its own limitations. A typical example is that the pile-up accumulating on the both sides of nanogroove fabricated by the TBN method on the polymer materials may cause a negative effect on the following etching process and the flow of the liquid in nanofluidic chips. Therefore, other methods should be integrated with the TBN method to generate more brand-new applications, which may push the TBN method on polymer materials technology toward industrialization in the near future.

## Figures and Tables

**Table 1:** The applications of scratching on polymers by the TBN method.

Theory	Polymer	Application	Refs.
	polymethyl methacrylate (PMMA) film	Etching resist	[52]
Remove material	polycarbonate (PC) bulk sample	Preparation of nanofluidic chips	[54]
	polymethyl methacrylate (PMMA) film	Data storage	[83]
Add material	polymethyl methacrylate (PMMA) film	Sacrificial layer for lift-off process	[53,78,95]
	polystyrene (PS) film	Sacrificial layer for lift-off process	[96]
Theothers	polycarbonate (PC) bulk sample	Resist for AFM (atomic force microscope) scratching	[97]
